# Active ankylosing spondylitis increases blood loss during total hip arthroplasty for a stiff hip joint

**DOI:** 10.1186/s12891-020-03278-2

**Published:** 2020-04-15

**Authors:** Yong Hu, Wei-Zhou Jiang, Cheng-Long Pan, Tao Wang

**Affiliations:** 1 Department of Orthopaedic Surgery, The Fifth Affiliated Hospital of Southern Medical University, Southern Medical University, Guangzhou, Guangdong Province China; 2 Department of Orthopaedic Surgery, NanFang Hospital, Southern Medical University, Guangzhou, China

**Keywords:** Ankylosing spondylitis, Blood loss, Disease activity, Total hip arthroplasty

## Abstract

**Background:**

Total hip arthroplasty (THA) has been highlighted as the best treatment option for ankylosing spondylitis (AS) patients with advanced hip involvement. The huge blood loss associated with THA is a common concern of postoperative complications. Disease activity is a specific reflection of systematic inflammation of AS. The purpose of this study was to determine the effect of disease activity on blood loss during THA in patients with AS.

**Methods:**

Forty-nine patients with AS who underwent unilateral THAs were retrospectively studied. Ankylosing Spondylitis Disease Activity Score (ASDAS) was employed to evaluate the disease activity. Orthopedic Surgery Transfusion Hemoglobin European Overview (OSTHEO) formula was used to assess the surgical blood loss. The patients were divided into active group (ASDAS≥1.3; *n* = 32) and stable groups (ASDAS< 1.3; *n* = 17) based on the ASDAS. Peri-operative laboratory values, plain radiographs, intra-operative data, transfusion volume, and use of hemostatic agents were recorded and statistically analyzed.

**Results:**

The ASDAS, pre-operative C-reactive protein level, erythrocyte sedimentation rate, and fibrinogen concentration in the active group were higher than the stable group (all *P* < 0.05); however, the pre-operative hemoglobin concentration and albumin level were higher in the stable group (both *P* < 0.05). The total blood loss during THA in stable patients was 1415.31 mL and 2035.04 mL in active patients (*P* = 0.006). The difference between the two groups was shown to be consistent after excluding the gender difference (*P* = 0.030). A high transfusion rate existed in both groups (stable group, 76.47% with an average of 1.53 units; active group, 84.37% with an average of 2.31 units), but there was no significant difference between the two groups (both *P* > 0.05). Compensated blood loss, corresponding to transfusion, was noted significantly more in the active group compared to the stable group (*P* = 0.027). There was no significant difference with regard to functional recovery (*P* > 0.05).

**Conclusion:**

Active AS patients are at high risk for increased blood loss during THA compared to stable patients. The underlying mechanism includes disorders of the coagulation and fibrinolytic systems, poor nutrition status, osteoporosis, imbalance of oxidative–antioxidative status and local inflammatory reaction. It is strongly recommended to perform THA in AS patients with stable disease.

## Background

Hip involvement is frequently reported in ankylosing spondylitis (AS), with a prevalence rates between 19 and 36% [1, 2]. For advanced ankylosis hip, total hip arthroplasty (THA) has been recommended as the best treatment option to correct deformities and improve joint function [3]. One study showed that the 10-year risks of THA was 2.6% for AS patients [4]. Another study reported that after more than 30 years’ disease 12–25% of patients had at least one replaced hip [1]. However, THA is usually associated with significant blood loss and high transfusion rate [5]. A systematic review of blood transfusion studies revealed that the allogenic transfusion rates ranged from 11 to 69% in THA surgery [6]. Moreover, AS leads to a bony ankylosis hip joint in the end stage. The operation will be much more difficult because the identification and exposure of the femoral neck and acetabulum are always complicated [7]. To reduce blood loss, most surgeons pay close attention to the surgical technique and use tranexamic acid, but rarely care the feature of disease [8–10].

Disease activity pointing to a feature of inflammation is believed to potentially affect the multi-system functions in AS. Significant correlations were found between work productivity, daily activity, psychological status and disease activity [11, 12]. Furthermore, disease activity was suggested as an increased risk of mortality [13], which might be associated with the impairment of cardiovascular system, coagulation-fibrinolytic system and microvascular function [14–16]. Abnormal bone metabolism was also common in AS [17, 18], and low bone mineral density was found to be associated with disease activity [19]. Generally, disease activity is deemed as a manifestation of underlying inflammation, which is closely correlated with vascular reaction, including angiotelectasis, accelerated flow, hyperemia, and exudation. Therefore, disease activity of AS may also influence the surgical blood loss of THA, which remains uncertain.

This retrospective cohort study is conducted to determine the effect of AS’s disease activity on the blood loss of THA. The current study aims to provide effective strategy to reduce the blood loss and make the operation safer.

## Methods

### Patients

Sixty-eight AS patients who underwent unilateral THAs between 2014 and 2018 in our department were retrospectively studied. The collected data included gender, age, height, weight, and disease duration. The exclusion criteria were as follows: other rheumatic diseases; active infection; tumors; and hematologic diseases, such as blood coagulation disorders, thrombocytopenia, and other hemorrhagic disorders. Patients with long-term use of anti-coagulants were also excluded.

Forty-nine patients (6 females and 33 males) were eligible for this study. The demographic characteristic are shown in Table 1. In 10 cases, biologics were used for patients with intolerable back pain until remission before THA [20]. No statistically significant differences existed between the stable and active disease groups with respect to age, gender, BMI, disease duration and biologics usage.

**Table 1:** Demographics and anthropometry of the study patients

	Stable disease group	Active disease group	P
Number of patients	17	32	
age (years)	31.65 ± 8.70	33.72 ± 11.92	0.531
Disease duration (years)	8.23 ± 2.86	12.41 ± 8.98	0.180^*^
BMI (kg/m^2^)	21.30 ± 2.40	21.33 ± 3.77	0.900^*^
Sex (female/male)	4/13	2/30	0.164^†^
Involved hip (left/right)	7/10	21/11	0.134^†^
Application of biologics (Y/N)	1/16	9/23	0.133^†^

^*^ non-parametric rank sum test, ^†^chi-square test with continuity correction, the rest- Student t-test

### Surgical protocol

Indications of THA among AS patients are refractory pain or disability and radiographic evidence of structural damage in the hips, regardless of age. All of the operations were performed using a standardized method by the same experienced team. Antibiotics were routinely administered intra-operatively and 24 h post-operatively. The hemostatic agents included hemocoagulase agkistrodon (HCA) and tranexamic acid (TXA). Combined intravenous and topical TXA were used during THA. Based on the guideline, combined venous thrombosis embolism (VTE) prophylaxis with mechanical and pharmacological methods were performed during hospitalization. Mechanical VTE prophylaxis was commenced at the same day after surgery. Anticoagulation drug was added at the first day after surgery when the patient’s risk of bleeding had been established as low. Extended-duration anticoagulation drug was used for up to 35 days following THA.

### Assessment

The following data were collected from patients: pre-operative indices, including C-reactive protein (CRP) levels, erythrocyte sedimentation rate (ESR), hemoglobin (Hb) concentration, hematocrit (Hct), platelet (Plt) count, and albumin (ALB) concentration; coagulation test indices (prothrombin time [PT], prothrombin activity [PT%], prothrombin time international ratio [PT-INR], activated partial thromboplastin time [APTT], thrombin time [TT], and fibrinogen concentration (Fbgc); post-operative Hct concentration; imaging feature; operative time; allogenic transfusion; autologous transfusion; and the strategy of hemostatic agent use. The preoperative plain radiograph of pelvis were collected to assess the bone quality of hip. The diagnosis of osteoporosis was determined by the classic grading of Singh’ index [21].

### Transfusion protocol

The transfusion triggers were based on the British guidelines [22] and clinical judgement. The traditional view on transfusion triggers was a Hb < 70 g/L and a hematocrit < 25%. A Hb < 80 g/L was a transfusion trigger for patients with cardiovascular and respiratory problems or patients > 65 years of age. Acute anemia, a drop in blood pressure (< 90/60 mmHg), dizziness, pale lips, weakness, and shortness of breath were also regarded as transfusion triggers.

### Calculation of blood loss

The intra-operative blood loss was estimated by weighing sterile cotton gauze pads and mostly by analysis of aspirated blood volume. However, in consideration the significant difference between estimated and calculated blood loss, the latter one was the main evaluation indicator [23]. Peri-operative blood loss was calcultaed by means of the Orthopedic Surgery Transfusion Hemoglobin European Overview (OSTHEO) formula [23, 24], as follows: total RBC loss (mL) = [uncompensated RBC loss (mL) + compensated RBC loss (mL)] / 0.35, uncompensated RBC loss (mL) = estimated blood volume (EBV) × (pre-operative Hct levels – post-operative Hct levels); EBV = (0.0235 × height (cm)^0.42246 × weight (kg)^0.51456) × k, where k = 2430 for women and 2530 for men; compensated RBC loss (mL) = allogenic erythrocyte units × 150 + autologous transfusion volume × 0.3.

Post-operative Hct levels were collected from the laboratory test that was carried out at 6 am 1 day after surgery. Only transfusions that were executed before phlebotomy for laboratory testing were included in the study.

### Evaluation of disease activity

AS disease activity was evaluated by the Ankylosing Spondylitis Disease Activity Score-CRP (ASDAS-CRP), a highly discriminatory index recommended by The Assessment in Ankylosing Spondylitis [25], as follows: ASDAS-CRP = 0.121 × back pain + 0.058 × duration morning stiffness + 0.110 × patient global + 0.073 × peripheral pain/swelling + 0.579 × Ln (CRP + 1). The following was suggested regarding ASDAS-CRP classification: ASDAS< 1.3, stable; 1.3 ≤ ASDAS< 2.1, moderately active; 2.1 ≤ ASDAS≤3.5, highly active; and ASDAS> 3.5, very highly active. In the present study, ASDAS< 1.3 was defined as stable disease and ASDAS≥1.3 was defined as active disease.

According to the classification of disease activity as we defined, more patients were assigned to the active group. Seventeen patients (35%) were in the stable group and 32 (65%) were in the active group. The average ASDAS in the stable group was 0.97 and 2.55 in the active group (*P* < 0.001)

### Complications

Five cases had post-operative complications during the 1st year after THA, and they were all in active disease group. Three patients suffered periprosthetic fracture, caused by accidentally fell down. The other 2 cases had dislocation. Other complications such as implant loosening, prosthetic joint infection, and deep venous thrombosis were not found.

### Statistical methods

All statistical analyses were performed using SPSS for Windows (version 23.0). Continuous data were presented as the mean ± standard deviation (SD). Comparisons of quantitative variables were performed using an unpaired Student t-test. In the case of heteroscedasticity, the non-parametric rank sum test was used. A chi-square test was executed for comparisons of qualitative variables. Differences at a level of *P* < 0.05 were identified statistically significant.

## Results

### Laboratory values, radiographs, operative time, and the use of hemostatic agents

**Fig. 1:**
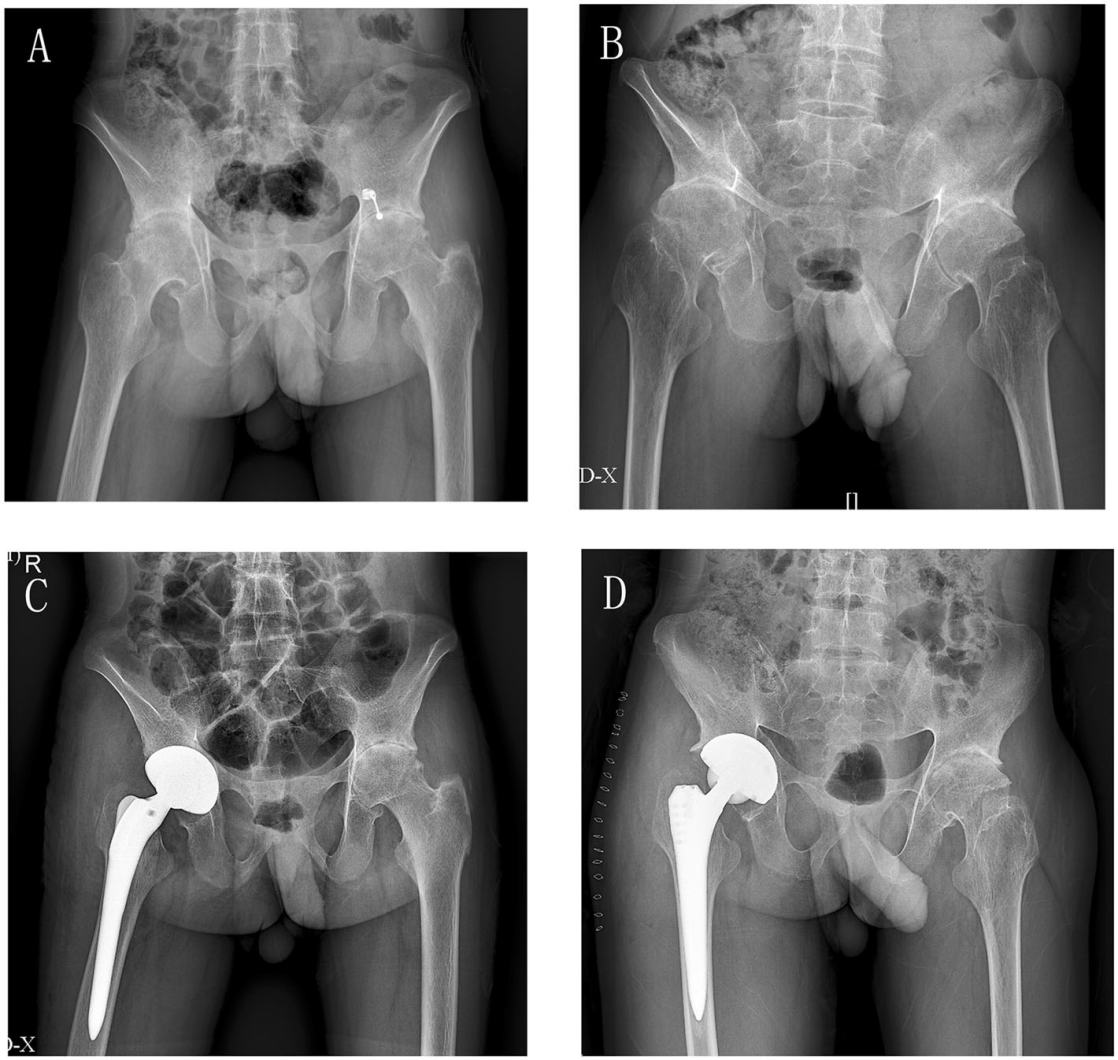
Radiograph features of patients with advanced hip lesions in ankylosing spondylitis who underwent primary cementless total hip arthroplasty (**a** and **c** for stable disease; **b** and **d** for active disease). For A and B, near complete fusion of the sacroiliac joints with erosive changes at the pubic symphysis were observed. Hip involvement is generally bilateral and symmetric, with significant joint space narrowing, reduced density of trabecular bone, cortical thinning and osteophytes

The pre-operative laboratory values, including CRP, ESR, and Fbgc in the active group, were significantly higher than the stable group (all *P* < 0.05); however, Hb and ALB were higher in the stable group (both *P* < 0.05). The coagulation indices, including PT, PT%, PT-INR, APTT, and TT, showed no statistical difference between the two groups. With respect to the operative time and the use of hemostatic agents, no significant difference was found between the two groups. The main results are shown in Table 2. The prevalence of osteoporosis assessed by imaging show no significant difference between the two groups. The typical radiographs were presented in Fig. 1.

**Table 2:** Laboratory values, ASDAS, operation time and hemostatic

	Stable disease group	Active disease group	P
ASDAS	0.97 ± 0.20	2.55 ± 0.90	< 0.001^*^
Preoperative CRP (mg/L)	3.04 ± 1.30	16.59 ± 11.91	< 0.001^*^
Preoperative ESR (mm/1 h)	10.82 ± 5.92	30.37 ± 20.16	< 0.001^*^
Preoperative Hb(g/L)	142.18 ± 13.03	132.41 ± 16.50	0.040
Preoperative Fbgc(g/L)	3.29 ± 8.18	3.77 ± 0.74	0.045
Preoperative ALB (g/L)	40.40 ± 2.68	38.59 ± 4.06	0.048^*^
Preoperative Hct (L/L)	0.421 ± 0.033	0.406 ± 0.041	0.200
Preoperative Plt(× 10^9^/L)	246.00 ± 67.86	277.62 ± 80.79	0.176
Preoperative PT(s)	12.48 ± 0.97	11.90 ± 1.07	0.071
Preoperative PT%	85.04 ± 13.03	92.65 ± 17.45	0.122
Preoperative PT-INR()	1.08 ± 0.10	1.03 ± 0.10	0.083
Preoperative APTT(s)	33.63 ± 3.09	32.61 ± 3.19	0.122
Preoperative TT(s)	17.22 ± 1.51	16.97 ± 1.95	0.651
Operation time (min)	93.53 ± 18.83	97.47 ± 32.80	0.925^*^
Osteoporosis in imaging (Y/N)	11/6	28/4	0.067^†^
The strategy of using hemostatic(a/b/c/d)	7/3/4/3	14/8/3/7	0.585^†^

^*^ non-parametric rank sum test, ^†^chi-square test with Fisher’s exact test, the rest- Student t-test;

a- “neither of HCA or TXA was used” b- “only HCA was used”,

c- “only TXA was used” d- “both of HCA and TXA were used”

### Blood loss and transfusion

The active group had approximately 600 mL more blood loss compared to the stable group, which was statistically significant (*P* = 0.006). The blood loss was 1415.31 ± 552.37 mL when AS was at the stable disease stage, while blood loss reached 2035.04 ± 791.35 mL at the active disease stage. The error potentially brought by the gender difference was also compared with the blood loss in males of both groups. We found that more blood loss was present in the active group consisting of all males than in the stable group with all males (*P* = 0.030). The change in Hct and uncompensated blood loss were not statistically different between the two groups.

The active group needed an additional 0.8 units of allogenic blood volume than the stable group, although no significant difference was found between the two groups (*P* = 0.066). Compensated blood loss, corresponding to transfusion, was noted significantly more often in the active group compared to the stable group (*P* = 0.027). A high ratio of transfusion was found in both groups (76.74% in the stable group and 84.37% in the active group); there was no statistically significant difference between the stable and active groups. Table 3 shows the results.

**Table 3:** Estimation of blood loss and allogenic transfusion

	Stable disease group	Active disease group	P
Change of Hct (L/L)	0.062 ± 0.035	0.081 ± 0.034	0.076
Uncompensated RBC loss (mL)	255.89 ± 149.08	327.32 ± 151.28	0.121
Compensated RBC loss (mL)	239.47 ± 169.92	384.94 ± 230.94	0.027
Total blood loss (mL)	1415.31 ± 552.37	2035.04 ± 791.35	0.006
Total blood loss (mL) (males)	1497.44 ± 570.95	2058.69 ± 811.94	0.030
Allogenic transfusion rate	13/17 (76.47%)	27/32 (84.37%)	0.700^†^
RBC transfusion(u)	1.53 ± 1.07	2.31 ± 1.53	0.066

^†^chi-square test, the rest- Student t-test;

### Post-operative functional recovery

Significant improvement in terms of symptoms and function was noted in both groups at one-year follow-up. However, there was no significant difference between the stable and active group. The results were showed in Table 4.

**Table 4:** Comparison between the two groups with regard to one-year functional recovery

	Stable disease group (*n* = 17)	Active disease group (*n* = 32)	P
Preoperative VAS	5.34 ± 1.72	6.30 ± 2.08	0.110
Postoperative VAS	2.34 ± 1.53	2.51 ± 1.69	0.724
Preoperative ROM			
Hip ankylosis	13 (76%)	25 (78%)	0.582^†^
The rest ROM in flexion(°)	33.75 ± 7.5	39.36 ± 6.82	N
The rest ROM in extension(°)	8.75 ± 4.78	9.71 ± 2.98	N
Postoperative ROM			
ROM in flexion(°)	79.41 ± 12.85	81.41 ± 12.52	0.601
ROM in extension(°)	14.71 ± 3.29	14.38 ± 4.35	0.785
Preoperative HHS	32.90 ± 7.98	29.59 ± 8.96	0.208
Postoperative HHS	88.68 ± 3.62	88.03 ± 4.39	0.604

*VAS* visual analog scale, *ROM* range of motion, *HHS* Harris hip score

^†^chi-square test, the rest- Student t-test;

## Discussion

As expected, we found more than 600 mL on average was found in the active disease group than the stable disease group *(P* = 0.006). The blood loss volume was 1415.31 mL at stable disease status, which even reached 2035.04 mL with disease activation. Jia et al. [5] reported that the average blood loss during THA reached 1517 mL for AS with bony ankylosis of the hips (ASB), which was exactly in the range between the volume of the stable and active groups in our study. Influential factors, such as surgical technique, bipolar electrocoagulation hemostasis technique, and the severity of hip ankylosis, might have affected the outcome. We suggested that the disease activity of AS might be another important influential factor correlated with the surgical blood loss because disease activity is easily ignored by surgeons. This issue was not considered by Jia et al. [5] and Zhao et al. [26].

In addition, the high transfusion rate in both groups (76.47% in the stable group and 84.37% in the active group) attracted our attention, which was consistent with the previous study [5]. It demanded a greater need to receive transfusion for AS patients undergoing THA. Averagely 2.31 units allogenic RBC was needed for active AS patients. Besides, the compensated blood loss that was positively correlated with allogenic transfusion was found more when AS was at the active disease status. The risk of transfusion reaction increases with the high allogenic transfusion rate; e.g., infection, alloimmunization and transfusion-related acute lung injury [27]. Therefore, disease activity of AS should be raised enough concern before surgery.

Our study discovered the emerging of Fbgc with disease activation, suggesting the influence of fibrinolysis system. Beinsberger et al. [28] proposed that the combined activation of platelets, leukocytes and endothelium affected the coagulation, fibrinolysis and immunity-induced procoagulation in the plasma. The plasma levels of key inflammatory mediators (ESR, CRP. TNF-α, IL-6) played an important role in the above activation [28]. In the synovial fluid, So et al. [15] also suggested the activation of coagulation and fibrinolysis system at active disease status of AS. We hypothesized an unbalance of coagulation and fibrinolysis activation locally with active disease, thereby clotting factors consuming and fibrinogen accumulation. A shortage of clotting factors might be attributed to the constant activation of the endogenous coagulation cascade reaction and the subsequent fibrinolysis reaction. The study of Pratic et al. proved a correlation between the delay of thrombin generation and disease activity in AS [29]. In that occasion, invasive operation was likely to increase blood loss.

Unlike the rise of Fbgc, we noticed that the pre-operative Hb and ALB concentrations were lower in the active group. It suggested that the poor nutrition status might be partially responsible for increased blood loss. Anemia and hypoproteinemia are common complications of chronic disease, and anemia is proven to be related to disease activity of rheumatoid arthritis [30]. Some researchers have reported that the morbidity of anemia in AS is 6% ~ 25%. Braun et al. [31] declared that the occurrence of anemia was associated with TNF-α in AS patients. Therefore, the immune inflammatory reaction might be responsible for the poor nutrition status. TNF-α, with an intensive biological effect, is regarded as the most important factor in the development of AS and proven to be positively associated with disease activity [32, 33]. We suggested the poor nutrition might also attributed to psychological disorders [12, 34]. The global function might be affected after long-term low nutritional condition and inflammatory cytokine activation. For example, the poor nutrition status is closely correlated with weak capability of repair, which might partially account for the increase in blood loss.

A high prevalence of osteopenia or osteoporosis in total hip was reported in AS [35–37]. Capaci et al. [35] found that 91.7% AS patients with advanced hip involvement suffered from osteoporosis. Limited by a retrospective study design, the current study employed the plain radiograph rather than dual-energy X-ray absorptiometry to assess the bone mass in total hip. The result was consistent with the previous report [35–37], with 64.7% patients who were diagnosis as osteoporosis in stable group and 87.5% patients in active group. Maillefert et al. [38] suggested that persistent inflammation might be an etiologic factor of bone loss in AS. Grazio et al. [39] and Wang et al. [40] further proposed that active disease accelerated bone loss in AS. However, our result denied the relationship between the disease activity and the prevalence of osteoporosis. The inconsistence might be attributed to the low sensitivity of plain radiographs to assess bone mass. During THA, the acetabula were more likely to be over-reamed and the risk of proximal femur fracture increased because of osteoporosis [7]. Zhao et al. [26] suggested that the osteoporosis might contribute to more blood loss due to periprosthetic microfracture. The impact of osteoporosis on surgical blood loss needs further discussion.

The study reported by Karakoc et al. [41] proposed a much higher level of total oxidative status level and oxidative stress index values, while the total antioxidative status level was lower in patients with AS. They further proved that some of the oxidants, such as nitric oxide and superoxide anion, from activated polymorphonuclear leucocytes were correlated to disease activity [41]. One of the most important biological effects of these oxidants is local hemangiectasis and accelerated blood flow. Therefore, the imbalance of oxidative–antioxidative status might also contribute to the increased blood loss.

In addition, local vascular damage may have been caused by oxidative stress and lipid peroxidation with disease activation, which leads to increased capillary permeability. It has been shown by Azevedo et al. [42] that interleukin-8, one of the biomarkers of endothelial damage, was higher in AS patients and the levels were clearly correlated with disease activity. Except for damage, angiogenesis also plays an important role in periarthritis of hip. Higher serum vascular endothelial growth factor (VEGF) levels was found in AS patients and positively correlated with disease activity [43]. Taken together, the periarthritis of hip includes acute and chronic inflammatory reaction when AS is in an active state, being featured with local hemangiectasis, accelerated blood flow, capillary permeation and angiogenesis. The consecutive reaction caused by active disease could be the main mechanism underlying increased blood loss. In our clinical practice, the inner incision was obviously observed much more errhysis during THA for AS patients.

The main limitations of this research are as follows: first, the number of cases might be small (49 hips) and the current study only enrolled Chinese population as the research cohorts. Racial difference might exist because AS was associated with various major histocompatibility complex genes, such as HLA-B27, HLA-B60 and HLA-DRB1 [44, 45]. Second, our findings should be further validated using a well powered prospective study with larger samples.

## Conclusion

In conclusion, active AS patients are at high risk for increased blood loss during THA compared to stable AS patients. The active group had approximately 600 mL more blood loss on average compared to the stable group. The underlying mechanism includes disorders of the coagulation and fibrinolytic systems, poor nutrition status, osteoporosis, imbalance of oxidative–antioxidative status and local inflammatory reaction. As blood loss increases, the length of hospital stay, post-operative rehabilitation, and the risk of incision infection might be affected. Thus, in consideration of blood loss, AS patients are recommended to undergo THA when in the stable disease stage. For active AS patients, efficient therapies should be applied to reduce disease activity to the stable level, of which TNF inhibitors, including adalimumab, etanercept, golimumab, and infliximab, are the first choices [46].

## Data Availability

The datasets used and/or analysed during the current study are available from the corresponding author on reasonable request.

## Abbreviations

THA: Total hip arthroplasty
AS: Ankylosing spondylitis
HCA: Heamocoagulase agkistrodon injection
TXA: Tranexamic acid injection
ASDAS: Ankylosing Spondylitis Disease Activity Score
CRP: C-Reactive protein
ESR: Erythrocyte Sedimentation Rate
Fbgc: Fibrinogen concentration
BMI: Body Mass Index
ALB: Albumin
PT: Prothrombin Time
PT%: Prothrombin activity
PT-INR: Prothrombin time international ratio
APTT: Activated partial thromboplastin time
TT: Thrombin time
Hb: Hemoglobin
Plt: Platelet
Hct: Hematocrit
